# Evaluation of PDZD11 in hepatocellular carcinoma: prognostic value and diagnostic potential in combination with AFP

**DOI:** 10.3389/fonc.2025.1533865

**Published:** 2025-03-25

**Authors:** Yiyun Ni, Bin Liu, Weizhen Zhang, Yilin Pang, Yaling Tian, Qingsong Lv, Shengwen Shi, Yang Zheng, Huihui Fan

**Affiliations:** ^1^ The Central Hospital of Yongzhou, Yongzhou Clinical College, University of South China, Yongzhou, Hunan, China; ^2^ School of Laboratory Medicine and Life Sciences, Wenzhou Medical University, Wenzhou, Zhejiang, China

**Keywords:** hepatocellular carcinoma, PDZD11, AFP, serological biomarker, prognosis

## Abstract

**Background:**

Hepatocellular carcinoma (HCC) is the most prevalent liver cancer, with a 5-year survival rate below 20% and an average survival time of 3-6 months. Identifying new biomarkers is crucial for early diagnosis and prognosis. The function of PDZ domain protein 11 (PDZD11) in HCC remains unclear.

**Methods:**

In this study, PDZD11 was investigated as a potential biomarker for HCC using bioinformatic analysis of the TCGA and ICGC datasets. Furthermore, we assessed the potential of serum PDZD11 as a clinical diagnostic marker by enrolling a cohort comprising 78 HCC patients and 62 healthy controls (HC) using the ELISA analysis and combining its expression with common tumor markers.

**Results:**

Our research found significantly higher PDZD11 mRNA expression in HCC tissues compared to tumor-adjacent tissues (*p* < 0.001), which was associated with lower overall survival (OS) rates (*p* < 0.01). Multivariate evaluation methods established PDZD11 as a standalone predictor of prognosis. A nomogram incorporating PDZD11 expression and clinicopathological factors predicted OS rates for HCC patients over various years. Patients with HCC exhibited notably elevated serum PDZD11 levels compared to HC, with these levels rising further in advanced disease stages and deteriorating performance status (PS). ROC analysis showed high diagnostic accuracy when PDZD11 is combined with AFP (AUC = 0.958).

**Conclusion:**

PDZD11 is more sensitive than AFP in assessing HCC prognosis. In conclusion, PDZD11 is a promising supplementary biomarker for HCC diagnosis and prognosis alongside AFP.

## Introduction

Liver cancer ranks as the sixth most common cancer globally and the third leading cause of cancer deaths, with hepatocellular carcinoma (HCC) comprising 80% of cases (1). Its incidence and mortality are increasing, particularly in East Asia and Africa (2, 3). Due to its prolonged incubation and rapid progression, over half of HCC cases are diagnosed at moderate to advanced stages, resulting in a five-year survival rate of only 18% (4, 5). Early-stage detection significantly improves outcomes, with over 70% of patients surviving at least five years post-surgery (6, 7). Thus, early diagnosis and careful monitoring are crucial for enhancing survival and reducing mortality.

In the early stages of HCC, imaging and serological tests are key diagnostic tools. Imaging techniques like CT and MRI have enhanced diagnostic accuracy but are costly, limiting their use for widespread screening (8). Liver biopsy, the gold standard for HCC diagnosis, is invasive and has a false negative rate of about 30% (9). This highlights the need for effective non-invasive biomarkers. Alpha-fetoprotein (AFP) is the most common serological marker, but its sensitivity and specificity depend on the threshold value. At lower thresholds, AFP has about 60% sensitivity for HCC, with suboptimal specificity, and over 30% of advanced HCC cases show normal AFP levels (10). Additionally, elevated AFP levels can occur in both benign and malignant conditions, including chronic hepatitis, liver cirrhosis, and certain cancers (11, 12). Recent studies highlight the potential of circulating biomarkers like AFP-L3, PIVKA-II, and others for noninvasive HCC screening (13–16). However, these biomarkers are still in pre-clinical stages, facing challenges like high costs and low specificity. Current research focuses on optimizing traditional markers and developing new blood-based markers (17–19). There’s a need for specific biomarkers to improve early HCC detection, prognosis assessment, and treatment prediction.

The PDZD11 protein, formerly known as PISP, AIPP1, and PDZK11, consists of 140 amino acids and 11 conserved PDZ domains, each with 2 α-helices and six β-sheets. It is found in the extracellular space, membrane, and cytoplasm, playing crucial roles in cellular functions like membrane sorting, copper balance, biotin absorption, and cell adhesion (20–25). Previous research shows that elevated *PDZD11* mRNA and protein levels in liver cancer correlate with reduced overall survival (OS) in HCC patients and increased immune cell infiltration (26). *PDZD11*, which enhances cell adhesion, is highly expressed in HPV16^+^ macrophages and positively correlates with cervical cancer patient survival (27). In epithelial ovarian cancer, PDZD11 is linked to cell adhesion, proliferation, and immune infiltration, and is upregulated, indicating a poor prognosis (28). Thus, PDZD11 is a potential biomarker for cancer diagnosis and prognosis.

Nevertheless, the clinical implications of PDZD11 in HCC are not yet fully understood. This study investigated PDZD11 expression by analyzing datasets from The Cancer Genome Atlas (TCGA) and the International Cancer Genome Consortium (ICGC) to assess its potential utility in the diagnosis and prognostication of HCC. Additionally, we explored the association between serum PDZD11 levels and the clinical and pathological characteristics of patients with HCC and healthy controls (HC). Furthermore, we assessed the diagnostic accuracy of PDZD11 both independently and in conjunction with AFP and examined its role in the progression and prognosis of HCC.

## Materials and methods

### Data collection and analysis

PDZD11 gene expression data and clinical information for HCC patients were retrieved from TCGA through the GDC data portal (https://portal.gdc.cancer.gov/) (29), including 425 samples (50 paracancerous and 375 tumor tissues). PDZD11 expression and clinical data from the ICGC dataset (https://dcc.icgc.org/) were also used to verify survival analysis. Perl was used to sort and merge gene expression data, while R’s “limma” package (version 4.2.3) extracted PDZD11 expression data. The “limma” and “beeswarm” packages visualized this data with scatter plots. Perl also extracted survival data, removing incomplete entries, and matched complete survival information with PDZD11 data, resulting in data for 370 eligible patients.

### Prognostic analysis

HCC patients were divided into high or low PDZD11 expression groups based on the median expression level. The Kaplan-Meier method evaluated variations in OS within the TCGA HCC group. The ‘survival’ package handled statistical analysis, while the survminer mapped survival curves. The receiver operating characteristic (ROC) analysis assessed the prediction accuracy and specificity of PDZD11. Additionally, key prognostic indicators were identified using univariate and multivariate Cox regression analysis with the ‘survival’ package in R (version 4.2.3).

### Development and assessment of clinical nomogram

We developed a nomogram for forecasting patient outcomes based on a multivariate Cox regression analysis. To evaluate the nomogram’s predictive accuracy, we also constructed a calibration plot. Both the nomogram and the calibration plot were generated using the ‘survival’ and ‘rms’ packages in R (version 4.2.3) (30).

### Study subjects and blood sampling

The study enrolled 78 patients newly diagnosed with HCC and 62 HC from the Central Hospital of Yongzhou. There were two categories of participants: HCC and HC. Diagnosis of HCC was confirmed based on China’s 2022 Guidelines for Primary Liver Cancer (31). Exclusion criteria included the presence of benign or malignant tumors in other locations, as well as other uncontrollable conditions such as severe infections, renal failure, and heart failure. The HC group underwent standard biochemical and immunological assessments to exclude the presence of hepatitis, cirrhosis, liver, gallbladder, and biliary tract tumors, as well as benign and malignant tumors in other regions. The research protocol received approval from the Ethics Committee of the Central Hospital of Yongzhou. This research followed the guidelines set forth in the Declaration of Helsinki and its amendments.

Blood samples were collected from each subject before surgery or treatment, subsequently centrifuged at 3,500 g for 10 min, and the supernatant was immediately frozen at -80°C, and the storage duration did not exceed six months.

### Collection of clinicopathological feature data

Information on clinicopathological characteristics, including factors like gender, age, tumor dimensions and quantity, cancer stage, lymph node status, distant spread, TBA, TBIL, DBIL, ALT, AST, WBC, and serum AFP levels, were meticulously gathered.

### Enzyme-linked immunosorbent assay measurements

PDZD11 protein concentrations were quantified utilizing a commercially available sandwich ELISA kit (EIAab, Wuhan, China), following the manufacturer’s protocol (https://www.eiaab.com.cn/). Each sample was assayed in duplicate. The intra- and inter-assay coefficients of variation were <5.2% and <9.6%, respectively.

### Statistical analysis

Data analysis utilized R (Version 4.2.3), SPSS (Version 20.0), GraphPad Prism (Version 9.5), and CurveExpert (Version 1.4). Normally distributed data were presented as means ± SD, and correlations between HCC and age were analyzed by the independent samples *t*-test. Non-normally distributed data were shown as medians and interquartile ranges (M [P25, P75]), and correlations between HCC and PDZD11, AFP, TBA, TBIL, DBIL, ALT, AST, and WBC were evaluated with the Mann–Whitney *U*-test. Categorical variables were presented using frequencies and percentages, and correlations between HCC and gender, Child-Pugh class, tumor size, tumor number, and tumor stage HBV or HCV were calculated by the Chi-square test. Diagnostic accuracy was assessed via ROC curve analysis, yielding AUC, threshold values, sensitivity, and specificity. Principal component analysis was conducted using the PCA expression plot feature in GraphPad Prism. Univariate logistic regression models were performed to test the association between PDZD11 and several clinical metrics, as well as the presence of HCC. Statistical significance was set at a *p*-value below 0.05.

## Results

### High PDZD11 expression in HCC indicates poor prognosis

**Figure 1:**
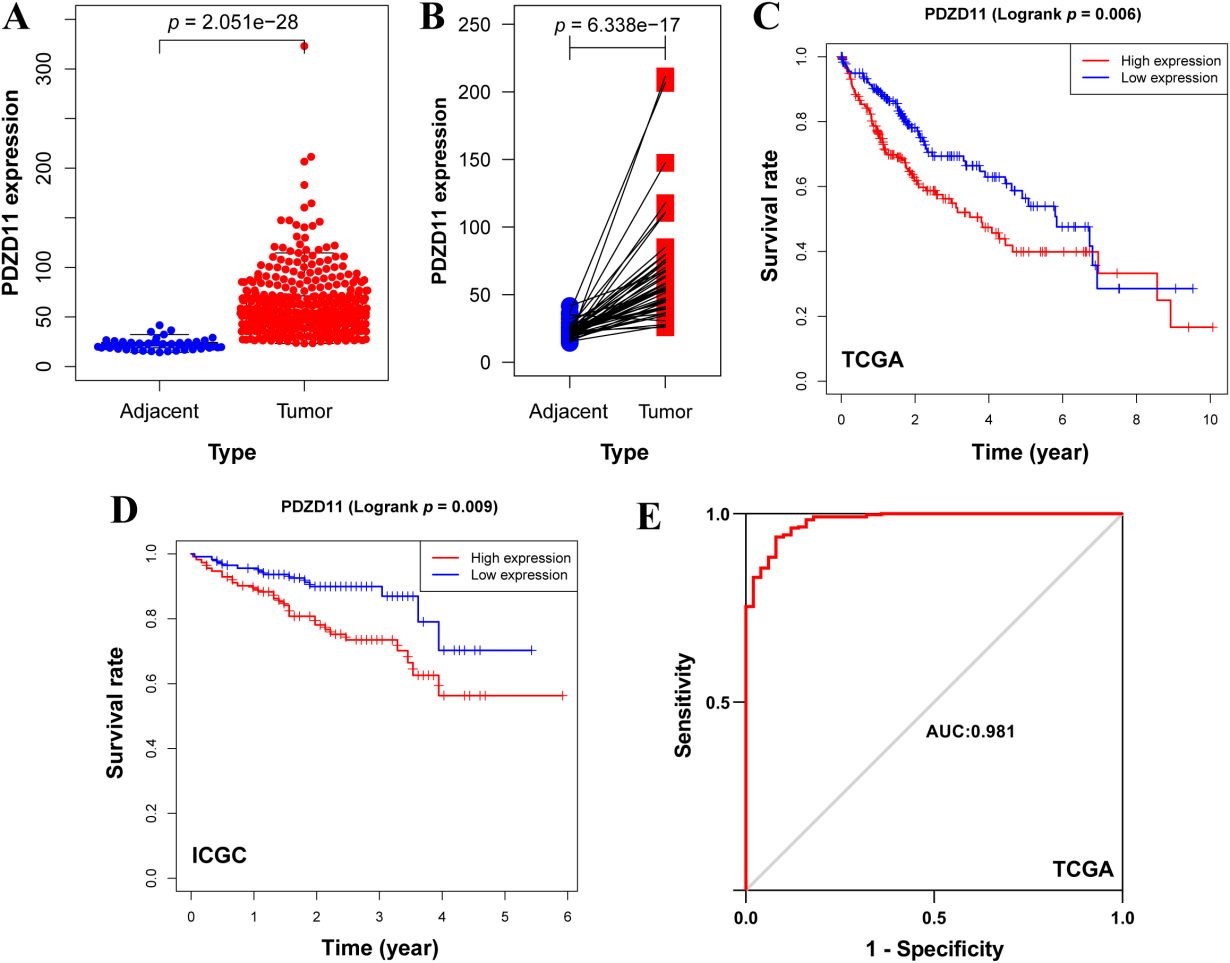
Elevated levels of *PDZD11* were indicative of a poor prognosis in HCC. **(A)** Boxplot depicting *PDZD11* expression levels in HCC versus adjacent tissues from the TCGA dataset. **(B)** Pairwise boxplot depicting the comparative expression of *PDZD11* between HCC and adjacent tissues in the TCGA dataset. **(C)** Kaplan-Meier survival analysis of *PDZD11* utilizing the TCGA dataset. **(D)** An analysis of the ICGC dataset to examine the effects of *PDZD11* expression in patients with HCC on OS. **(E)** The ROC curve illustrated the diagnostic value of *PDZD11* in HCC patients.

A Wilcoxon rank-sum test showed significantly higher *PDZD11* levels in HCC samples compared to adjacent ones (**Figure 1A**). By applying the Wilcoxon signed-rank test to 50 paired samples of liver cancer and adjacent tissues, we verified that *PDZD11* expression was markedly reduced in the adjacent tissues (**Figure 1B**). Kaplan-Meier analysis revealed that HCC patients with high *PDZD11* levels had shorter OS compared to those with low levels (*p* < 0.01) (**Figure 1C**). In the same way, analysis of the ICGC dataset verified that elevated *PDZD11* levels in HCC correlate with reduced OS (*p* < 0.01) (**Figure 1D**). The ROC curve analysis showed that *PDZD11* had an AUC of 0.981, indicating high accuracy (**Figure 1E**). This suggests PDZD11 could be a valuable biomarker for diagnosing and predicting HCC prognosis.

### PDZD11 might independently predict HCC prognosis

**Figure 2:**
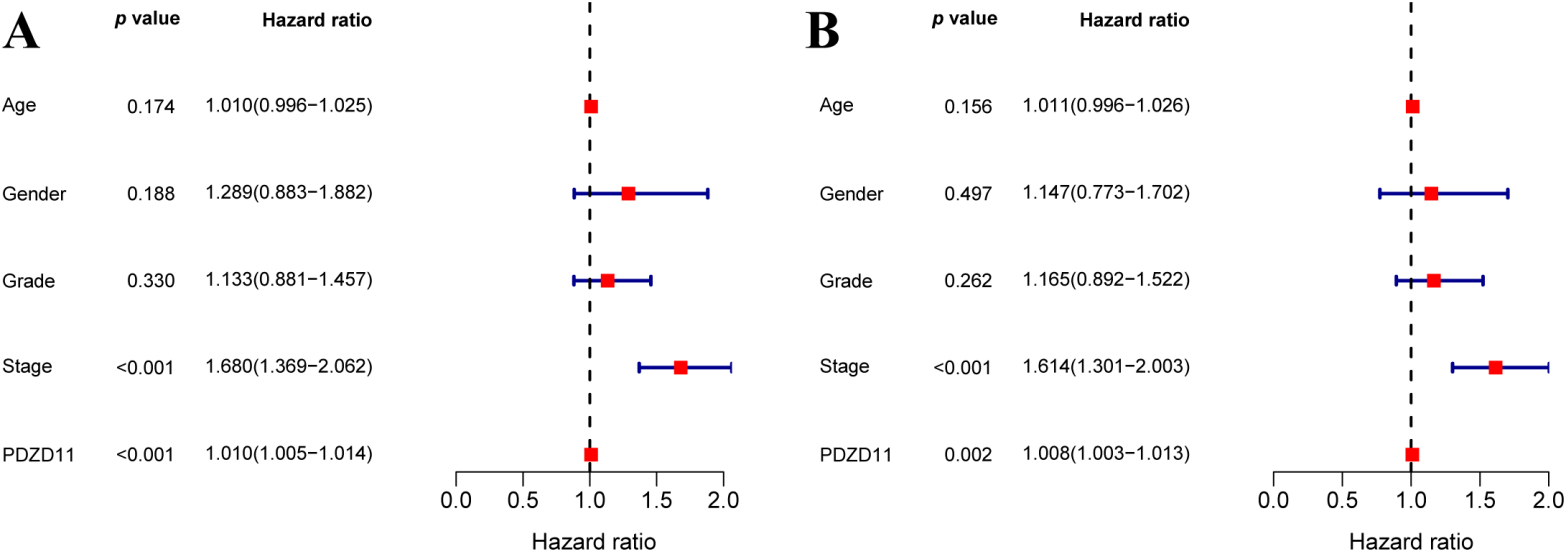
To explore the link between *PDZD11* levels and OS in HCC patients, both univariate **(A)** and multivariate **(B)** COX proportional hazards models were utilized.

We assessed predictors of OS through both univariate and multivariate Cox regression models (**Figure 2**). According to the univariate analysis, both cancer stage and *PDZD11* levels were significantly associated with OS in HCC patients (*p* < 0.001) (**Figure 2A**). According to multivariate analysis, both stage and *PDZD11* expression (all *p* < 0.01) were identified as independent prognostic indicators (**Figure 2B**).

### Nomogram creation using PDZD11 expression and clinicopathologic factors

**Figure 3:**
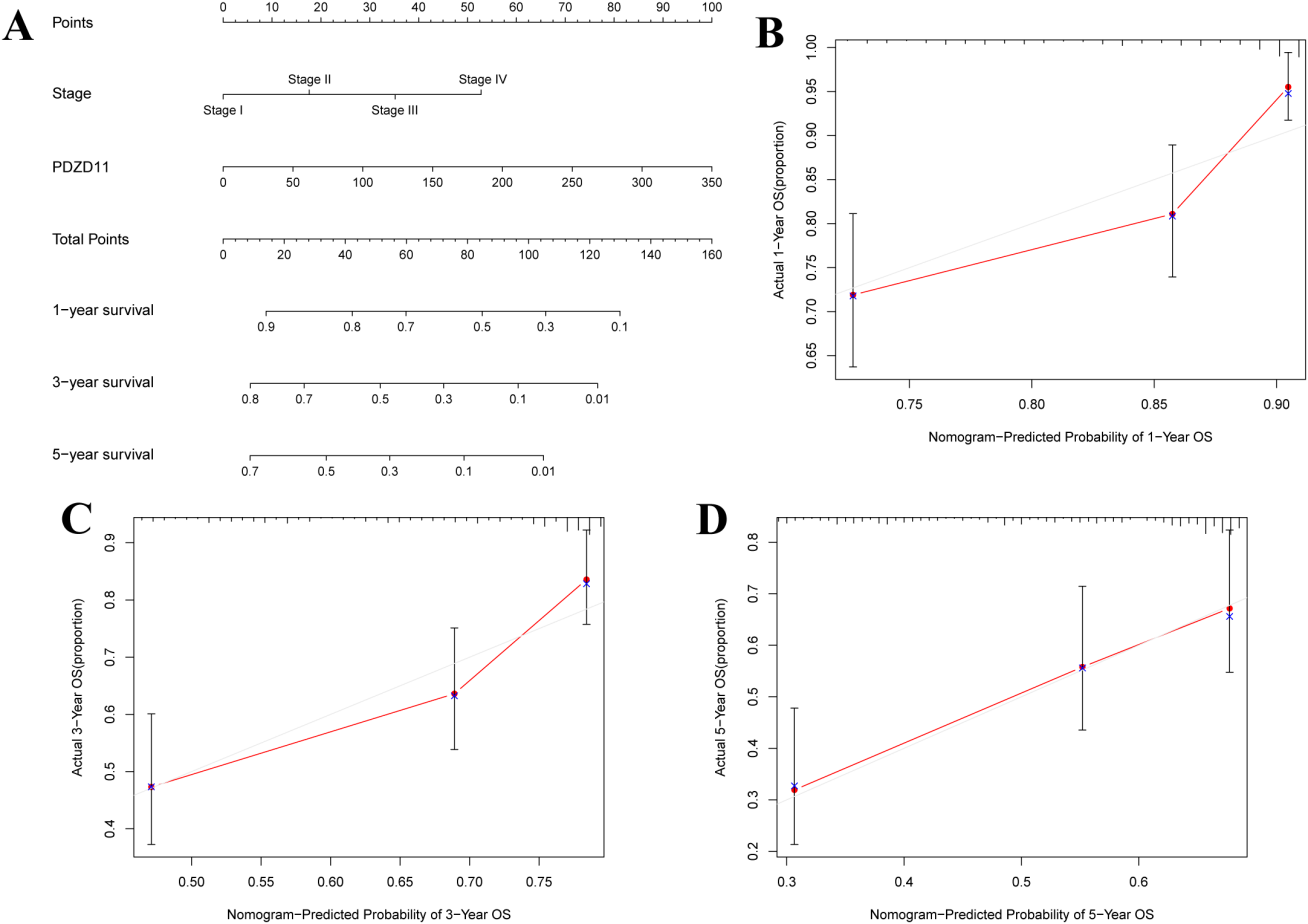
A nomogram for predicting OS in patients with HCC. **(A)** Development of a nomogram incorporating *PDZD11* and clinicopathologic variables. For each patient, two lines were drawn to get points from the predictors (stage and *PDZD11*) in the nomogram. These points were summed and located on the ‘Total Points’ axis. A line was then drawn downwards to determine the 1-, 3-, and 5-year overall survival probabilities for HCC. **(B)** Calibration curves for 1-year predictions. **(C)** Calibration curves for 3-year predictions. **(D)** Calibration curves for 5-year predictions. Nomogram-predicted survival could be visualized on the X-axis, while actual survival can be seen on the Y-axis.

A clinical tool was developed using a nomogram based on stage and *PDZD11* expression to predict 1-, 3-, and 5-year OS in HCC patients. **Figure 3A** illustrates that higher total points correlate with poorer survival outcomes. While the calibration plots for 1-year and 3-year predictions (**Figures 3B, C**) suggested potential underestimation or overestimation of mortality by the nomogram, the calibration plot for the 5-year predictions (**Figure 3D**) demonstrated satisfactory predictive accuracy, with the bias-corrected line closely matching the ideal curve, indicating a strong correlation between observed and predicted values. These results demonstrated a satisfactory performance of the constructed nomogram.

### The clinical and laboratory characteristics of HCC and HC

This study examined a cohort consisting of 78 patients diagnosed with HCC and 62 HC, as detailed in **Table 1**. Among the 140 participants enrolled, 128 (91.4%) were male and 12 (8.6%) were female. The mean ages for the HCC patients and HC were 58.8 ± 1.0 years and 59.3 ± 0.7 years, respectively. The two groups showed no notable variations in age or gender distribution. **Table 1** displays the initial traits of individuals with HCC and HC.

**Table 1:** Baseline characteristics of HCC patients and HC subjects.

Characteristics	HCC (%) n = 78	HC (%) n = 62	*p*-value
Age (years)			0.9524
30-50	14 (18.0)	3 (4.8)	
51-70	59 (75.6)	57 (91.9)	
> 70	5 (6.4)	2 (3.2)	
Gender			0.8524
Female	7 (9.0)	5 (8.1)	
Male	71 (91.0)	57 (91.9)	
PS			
0-2	67 (85.9)		
3-4	11 (14.1)		
Child-Pugh class			
A	41 (52.6)		
B	28 (35.9)		
C	9 (11.5)		
Tumor size (cm)			
≥ 5 c m	54 (69.2)		
< 5 c m	24 (30.8)		
Tumor number			
Single	13 (16.7)		
Multiple	65 (83.3)		
Tumor stage			
I-II	7 (9.0)		
III-IV	71 (91.0)		

### Correlation between serum PDZD11 levels and clinicopathological features in HCC patients

**Figure 4:**
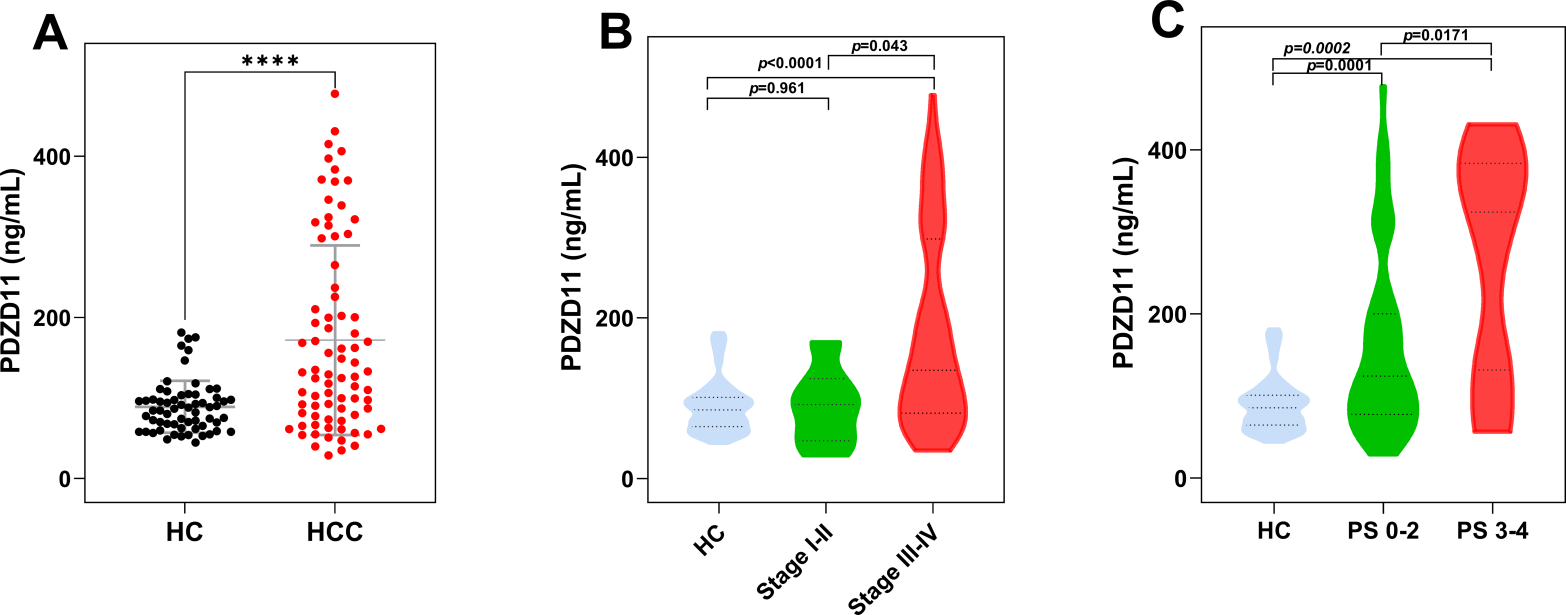
The expression levels of serum PDZD11 in HCC by subgroup. **(A)** Serum PDZD11 levels in HCC and HC groups. **(B)** Serum PDZD11 levels across various clinical stages of HCC. **(C)** Serum PDZD11 levels in HCC patients stratified by performance status. Statistical significance was used by the Mann–Whitney U-test. ****p < 0.0001.

The ELISA analysis for PDZD11 was conducted on a cohort comprising 78 HCC patients and 62 HC. Serum PDZD11 levels of HCC patients were markedly higher than those in HC (*p* < 0.0001, **Figure 4A**). In particular, the median serum levels of PDZD11 were 131.72 ng/ml (range: 77.42–264.86 ng/ml) in HCC patients and 85.45 ng/ml (range: 64.59–100.9 ng/ml) in HC. The results indicate that PDZD11 could act as a possible blood-based marker for HCC. Nonetheless, there were no notable associations between serum PDZD11 concentrations and factors such as age, sex, tumor dimensions, tumor count, or Child-Pugh classification (*p* > 0.05). Additional examination showed that patients with stage III-IV tumors had increased serum PDZD11 protein levels compared to those with stage I-II tumors (*p* = 0.043), and these levels were notably higher than in the HC group (*p* < 0.0001, **Figure 4B**). On the other hand, there was no notable disparity between the HC group and patients with stage I-II tumors (*p* = 0.961, **Figure 4B**). Notably, there was a higher correlation between serum PDZD11 levels and poor performance status (PS) in HCC patients (ECOG PS 3-4) compared to those with good PS (ECOG PS 0-2) (*p* = 0.0171, **Figure 4C**). Furthermore, both the poor and good PS groups had markedly higher serum levels of PDZD11 compared to the HC group.

### PDZD11 as a potential serological biomarker for diagnosing and prognosing HCC

**Figure 5:**
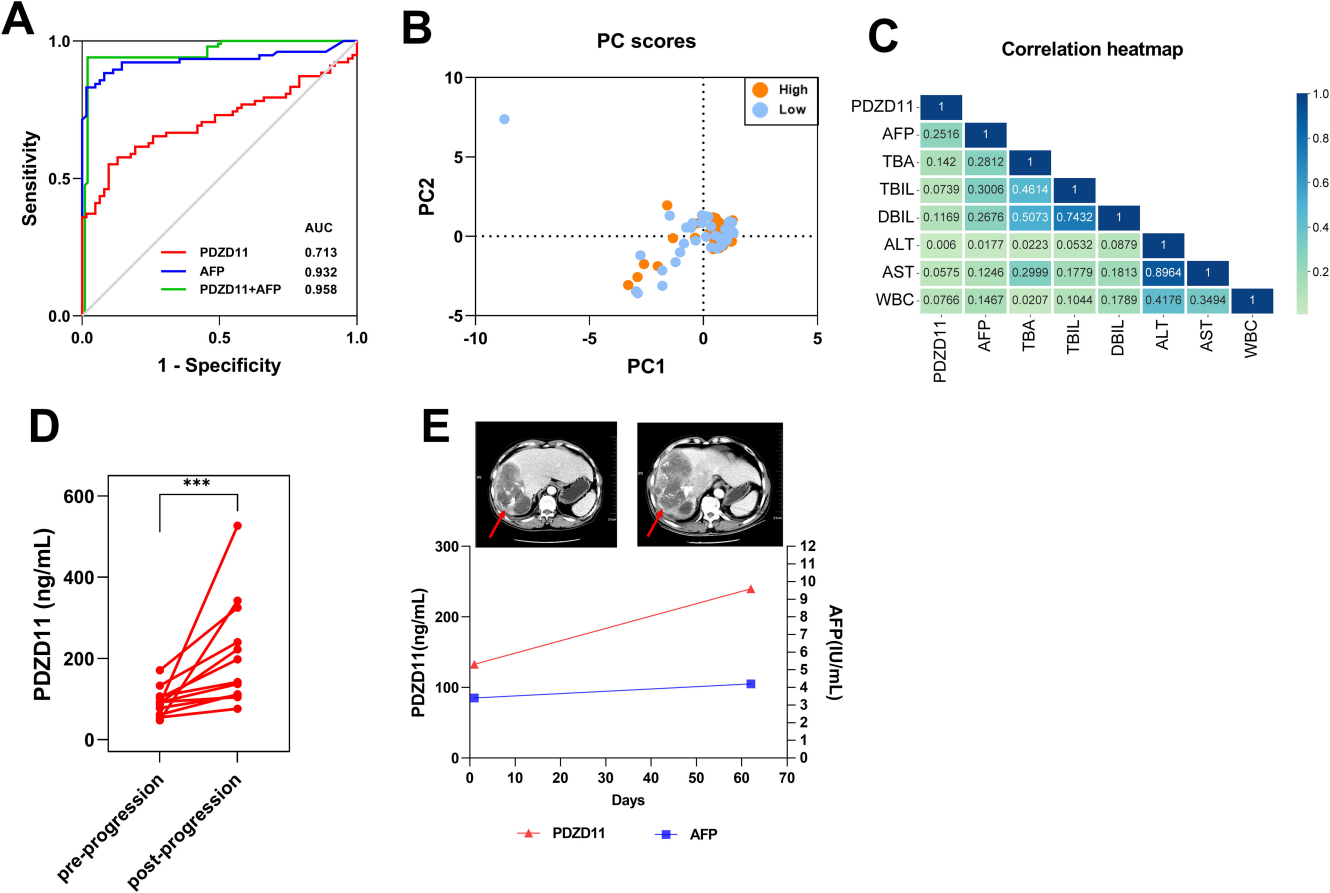
The role of serum PDZD11 levels in the diagnosis and evaluation of therapeutic response. **(A)** The performance of PDZD11 and AFP in ROC curve analysis for the diagnosis of HCC. **(B)** Analysis of clinical indices linked to PDZD11 expression using PCA in groups with high and low expression levels. **(C)** Univariate logistic regression analysis of PDZD11 levels and clinical indices. **(D)** Comparison of serum PDZD11 levels pre- and post-progression. **(E)** Monitoring of the HCC patient using PDZD11 and AFP analysis in conjunction with the abdominal CT. The lesion on the abdominal CT was indicated by a red arrow. ***p < 0.0001.

To assess PDZD11 and AFP’s diagnostic performance as individual biomarkers, a ROC curve was constructed. As illustrated in **Table 2** and **Figure 5A**, both markers demonstrated significant diagnostic efficacy in differentiating HCC from HC, with PDZD11 achieving an AUC of 0.713 (95% CI: 0.626 - 0.799) and AFP achieving an AUC of 0.932 (95% CI: 0.884 - 0.979). Youden’s index was employed to ascertain the optimal threshold values for PDZD11 and AFP. For PDZD11, the optimal threshold for distinguishing HCC from HC was 105.50 ng/mL, corresponding to a sensitivity of 0.615 and a specificity of 0.807. For AFP, the optimal threshold for AFP was determined to be 6.085 ng/ml, yielding a sensitivity of 0.831 and a specificity of 0.984. Importantly, the capability to differentiate HCC from HC improved markedly, as the AUC for the PDZD11 and AFP combination hit 0.958, whereas AFP alone had an AUC of 0.932 (*p* < 0.0001, **Figure 5A**). Moreover, the sensitivity of the diagnosis using the combined markers (PDZD11 + AFP) increased to 0.941, compared to 0.831 for AFP alone (**Table 2**).

**Table 2:** Performance of serological biomarkers for the diagnosis of patients with HCC.

Markers	AUC (95%CI)	*p-value*	Cutoff value	Sensitivity	Specificity	PPV	NPV
PDZD11	0.713 (0.626 - 0.799)	< 0.0001	105.500ng/ml	0.615	0.807	0.803	0.632
AFP	0.932 (0.884 -0.979)	< 0.0001	6.085 ng/ml	0.831	0.984	0.985	0.813
PDZD11+ AFP	0.958 (0.928 to 0.989)	< 0.0001		0.941	0.980	0.986	0.910

PPV, Positive predictive value; NPV, Negative predictive value. A *p*-value of < 0.05 was considered statistically significant.

HCC patients were categorized into high- and low-expression groups according to the level of PDZD11 protein expression (**Supplementary Table S1**). As illustrated in **Supplementary Table S1**, the findings indicated a significant association between PDZD11 expression and the Child-Pugh class (*p* < 0.0001). Conversely, an analysis using PCA on the PS score, tumor dimensions, tumor count, AFP, TBA, TBIL, DBIL, ALT, AST, and WBC revealed no notable distinctions between the two groups (**Figure 5B**). Furthermore, univariate logistic regression analysis demonstrated a weak association between HCC and PDZD11, AFP, TBA, TBIL, DBIL, ALT, and AST levels (**Table 3**, **Figure 5C**).

**Table 3:** The findings from the univariate logistic regression analysis of PDZD11 and several clinical metrics.

Valuable	Univariate analysis		*p*-value
	OR (Odds ratios)	95% CI	
Gender	1.124	0.341 to 3.973	0.8486
Age	0.991	0.948 to 1.034	0.6656
PDZD11	1.015	1.009 to 1.023	<0.0001
AFP	1.422	1.209 to 1.815	0.0005
TBA	1.237	1.144 to 1.366	<0.0001
TBIL	1.104	1.054 to 1.170	0.0002
DBIL	1.569	1.317 to 1.970	<0.0001
ALT	1.089	1.052 to 1.138	<0.0001
AST	1.345	1.212 to 1.555	<0.0001
WBC	0.991	0.895 to 1.093	0.8616

TBA, Total bile acid; TBIL, Total bilirubin; DBIL, Direct bilirubin; ALT, Alanine aminotransferase; AST, Aspartate transferase; WBC, White blood cell. A *p*-value of < 0.05 was considered statistically significant.

The PDZD11 levels of 12 patients, encompassing a total of 24 post-progression blood samples, were continuously monitored (**Figure 5D**). Additionally, we examined the clinical information of patients who had paired ‘before and after’ abdominal CT of tumor progression to evaluate the relationship between PDZD11 levels and tumor progression. For instance, one patient exhibited clinical progression as confirmed by abdominal CT two months after transarterial chemoembolization (TACE) combined with loplatin and pirarubicin interventional therapy. Although AFP levels remained within normal ranges both before and after progression, PDZD11 levels were significantly increased compared to pre-progression levels (**Figure 5E**). This suggests that PDZD11 may possess greater sensitivity than AFP in assessing the therapeutic response of HCC patients.

## Discussion

HCC is the most common among primary liver cancers. Major contributors to the onset of HCC include long-term alcohol use, exposure to aflatoxins, diabetes, obesity-induced non-alcoholic steatohepatitis (NASH), and persistent infections with hepatitis B or C viruses (HBV or HCV) (32). Because the initial signs of HCC are unusual, most patients are identified at intermediate to late stages, and just 30-40% qualify for surgery. Therefore, it’s crucial to increase survival rates in HCC patients by implementing screening guidelines for high-risk groups and ensuring early diagnosis (33). AFP is the most frequently utilized biomarker for patients with HCC. Combining serum AFP with other biomarkers boosts early-stage HCC diagnosis, making it a valuable approach for better detection rates (34).

PDZ domain proteins have recently gained recognition as biomarkers indicative of poor prognosis in HCC and as potential targets for immunotherapy (35). Our previous study investigated the prognostic significance and functional implications of PDZD11 in liver cancer using bioinformatics approaches. Furthermore, PDZD11 expression was significantly increased in liver cancer tissues and cell lines, implicating that PDZD11 play an important role in the progression of liver cancer (26). Currently, PDZD11 protein’s function in liver cancer is a mystery, and no extensive studies have explored serum PDZD11 levels in patients with liver cancer.

This study identified a significant elevation in PDZD11 expression in HCC tissues relative to adjacent non-cancerous tissues, as determined through analyses of the TCGA and ICGC datasets. Analysis of the ROC curve demonstrated that PDZD11 had excellent diagnostic precision in differentiating HCC from HC, achieving an AUC of 0.981. Additionally, HCC patients exhibited significantly elevated serum PDZD11 levels in comparison to HC. The diagnostic accuracy of PDZD11 was further enhanced when combined with AFP, yielding an AUC of 0.958. According to these findings, PDZD11 may serve as a reliable diagnostic biomarker for HCC, consistent with our prior research (26).

Furthermore, the Kaplan-Meier survival analysis revealed that higher levels of PDZD11 expression were associated with decreased OS in HCC patients. In multivariate Cox regression analyses, PDZD11 was confirmed to be an independent predictor of prognosis. Constructing a nomogram with PDZD11 expression and clinicopathological variables revealed a strong concordance between observed and predicted survival probabilities, underscoring its potential utility in personalized patient management. Additionally, serum PDZD11 levels were positively correlated with tumor stage and ECOG-PS scores, suggesting PDZD11 could be a prognostic biomarker. Specifically, higher clinical stages were observed in patients with poorer health status. The capacity of PDZD11 to assess therapeutic responses in HCC patients further highlights its potential clinical utility. Notably, serum PDZD11 levels were elevated following HCC progression compared to pre-progression levels, demonstrating its sensitivity in monitoring therapeutic responses.

The limitations of our study warrant further investigation. Firstly, the bioinformatics analysis has certain limitations, due to the small amount of PDZD11 expression data for HCC and adjacent non-cancerous tissues in the TCGA and ICGC datasets. Secondly, the small cohorts and short follow-up duration are quite limited for survival analysis, so additional validation studies with larger groups are needed to substantiate our results. Further research is required to elucidate PDZD11’s role in HCC development and assess its potential as a therapeutic target. Simultaneously, the specificity of AFP in diagnosing HCC in this study was determined to be 0.984. This contrasts with findings from other studies, which have reported AFP specificity in the range of 0.60 to 0.90 (36–38). The elevated specificity observed in our study may be attributable to the exclusion of a non-HCC disease cohort, a decision influenced by the context of hospitalized patients and temporal limitations. Consequently, this exclusion likely contributed to the higher specificity values for both AFP and PDZD11. Additionally, the diagnostic value and OR evaluation of PDZD11 were inferior to AFP. This may be attributed to factors such as the small sample size, the exclusion of non-HCC subgroups, and limitations in the detection methodology. Therefore, the clinical significance of PDZD11 protein in HCC deserves further investigation in future studies.

To conclude, PDZD11 shows considerable promise as a marker for diagnosing and predicting the outcome of HCC. Its clinical significance is highlighted by PDZD11’s elevated expression levels in both tissues and serum of HCC patients, along with its association with poor prognoses. This research is the first to indicate that PDZD11 shows potential as a supplementary biomarker for HCC, thus improving diagnostic accuracy. These findings offer a valuable understanding of the usefulness of PDZD11 in diagnosing and prognosis for HCC. However, in order to fully understand PDZD11’s clinical applications, as well as to further validate its efficacy in larger cohorts and elucidate its molecular mechanisms in HCC, further research is necessary.

## Data Availability

The raw data supporting the conclusions of this article will be made available by the authors, without undue reservation.
