# Supplementary material for: Evaluation of PDZD11 in hepatocellular carcinoma: prognostic value and diagnostic potential in combination with AFP
Source: Front Oncol. 2025 Mar 25;15:1533865. doi: 10.3389/fonc.2025.1533865 (PMC11975663; doi:10.3389/fonc.2025.1533865)
Supplement: Supplementary file 1 [file Table1.doc]

**Supplementary TABLE S1**  The correlation between serum PDZD11 protein expression and clinical indices in HCC patients.

| **Characteristics** | **PDZD11** **protein expression** | | ***p-*value** |
| --- | --- | --- | --- |
|  | **Low (n=39)** | **High (n=39)** |  |
| Gender |  |  | 0.4309 |
| Female | 5 | 2 |  |
| Male | 34 | 37 |  |
| PS |  |  | 0.5444 |
| 0-2 | 36 | 31 |  |
| 3-4 | 3 | 8 |  |
| Child-Pugh class |  |  | **< 0.0001** |
| A | 19 | 22 |  |
| B | 15 | 13 |  |
| C | 5 | 4 |  |
| Tumor size (cm) |  |  | 0.4622 |
| ≥ 5 c m | 25 | 29 |  |
| < 5 c m | 14 | 10 |  |
| Tumor number |  |  | 0.2235 |
| Single | 9 | 4 |  |
| Multiple | 30 | 35 |  |
| Tumor stage |  |  |  |
| I-II | 5 | 2 | 0.4309 |
| III-IV | 34 | 37 |  |
| HBV or HCV |  |  | > 0.9999 |
| Yes | 34 | 33 |  |
| No | 5 | 6 |  |
| Age | 57.03±10.12 | 60.54 ± 7.880 | 0.0912 |
| AFP (IU/mL) | 329.9 (10.70, 1000) | 132.3 (19.80, 1000) | 0.9579 |
| TBA (μmol/L) | 21.40 (10.20, 50.00) | 17.50 (5.90, 35.10) | 0.3355 |
| TBIL (mmol/L) | 27.81 (15.50, 56.98) | 22.53 (17.43, 40.06) | 0.8289 |
| DBIL (mmol/L) | 11.11 (6.06, 25.00) | 10.99 (7.44, 18.78) | 0.8953 |
| ALT (U/L) | 55.00 (26.50, 117.4) | 38.60 (20.00, 80.30) | 0.3560 |
| AST (U/L) | 76.90 (47.20, 213.1) | 58.60 (36.50, 101.50) | 0.0968 |
| WBC (× 10^9^/L) | 5.78 (4.45, 7.62) | 4.53 (3.25, 9.10) | 0.2657 |

Note: Abbreviations: TBA: Total bile acid; TBIL: Total bilirubin; DBIL: Direct bilirubin; ALT: Alanine aminotransferase; AST: Aspartate transferase; WBC: White blood cell. A *p*-value of < 0.05 was considered statistically significant.
